# Prediction of early recurrence of hepatocellular carcinoma within the Milan criteria after radical resection

**DOI:** 10.18632/oncotarget.18799

**Published:** 2017-06-28

**Authors:** Jiliang Feng, Junmei Chen, Ruidong Zhu, Lu Yu, Yan Zhang, Dezhao Feng, Heli Kong, Chenzhao Song, Hui Xia, Jushan Wu, Dawei Zhao

**Affiliations:** ^1^ Clinical-Pathology Center, Beijing You-An Hospital, Capital Medical University, Beijing, China; ^2^ Medical Laboratory Center, Beijing You-An Hospital, Capital Medical University, Beijing, China; ^3^ Surgical Center, Beijing You-An Hospital, Capital Medical University, Beijing, China; ^4^ College of Life Science, Sichuan University, Chengdu, China; ^5^ Surgical Center, The 304th Hospital of PLA, Beijing, China; ^6^ Medical Imaging Department, Beijing You-An Hospital, Capital Medical University, Beijing, China

**Keywords:** hepatocellular carcinoma, Milan criteria, radical resection, cytokeratin 19, glypican 3

## Abstract

Approximately 50% hepatocellular carcinoma patients meeting the Milan criteria utilized to develop an improved prognostic model for predicting the recurrence in these patients. Using univariate and multivariate analysis, cytokeratin-19 and glypican-3 expression patterns, tumor number and histological grading from eight putative prognostic factors comprised the risk factor scoring model to predict the tumor recurrence. In the training cohort, the area under roc curve (AUC) value of the model was 0.715 [95% confidence interval (CI) = 0.645−0.786, P<0.001], which was the highest among all the parameters. The performance of the model was assessed using an independent validation cohort, wherein the AUC value was 0.760 (95% CI=0.647−0.874, P<0.001), which was higher than the other factors. The results indicated that model had high performance with adequate discrimination ability. Moreover, it significantly improved the predictive capacity for the recurrence in patients with hepatocellular carcinoma within the Milan criteria after radical resection.

## INTRODUCTION

Hepatocellular carcinoma (HCC) is the most common type of liver cancer and is a leading cause of cancer-related death worldwide. When compared to the advanced HCC, patients at early stage usually have longer overall survival and disease free survival rates after treatment. While according to the previous reports, 50 percent of early stage HCC patients could recur within three years after radical resection (RR) [1, 2]. But currently the concept of early stage HCC remains little inconsistent due to the risk of recurrence after treatments varied depending on the defined thresholds of tumor diameters [3, 4, 5], however, majority of the patient groups were selected according to the Milan criteria (MC) which is based on the macro-morphological criteria [6].

A comprehensive evaluation of tumor helps in the determining the treatment modality. Currently, in most consensus criteria, only tumor size and numbers were considered in the evaluation of early stage HCC. But ongoing research provided identification of new and reliable molecular markers that were closely associated with the classification of tumors which may help improve the selection criteria process for HCC patients. In addition, majority of early recurrence of HCC was related to the intrahepatic or extrahepatic dissemination of the primary tumor, investigation of molecular markers related to the early recurrence of the tumor could help in the evaluation of prognosis and develop selection criteria for optimizing treatment strategies [7, 8].

Traditionally, HCC has long been believed to be transformed from the mature hepatocytes by dedifferentiation process [9]. With the understanding of the hierarchical makeup of parenchymal cells in both fetus and adult normal liver as well as in the chronic liver diseases, it was now realized that HCC consisted of a heterogeneous group of subtypes, which may have transformed from not only the hepatic progenitor cell (HPC), but also their progenies [10, 11]. Jiliang Feng *et al* based on the expression phase and spectrum of cytokeratin 19 (CK19) and glypican 3 (GPC3) had sub-classified HCC into CK19+/GPC3+, CK19−/GPC3+, and CK19−/GPC3−phenotypes, which roughly corresponded to HCC subtype transform from the HPC, immature hepatocyte, and terminal differentiated hepatocyte, respectively. And these patients showed the risk of intrahepatic metastasis, microvascular invasion, regional lymph node involvement, and distant metastasis were successively decreased. Their work implied that the cellular origin of differentiation was closely linked to the aggressive biological behavior of tumor cells [12].

Prognostic significance of CK19 or GPC3 in HCC had been largely investigated previously [13, 14, 15, 16]]. However, by combining CK19 and GPC3, a more precise sub-classification of HCC can be defined which might have produced a more efficient stratification for the prognosis of HCC patients. Hence, in the present study, a molecular indicator combining CK19 and GPC3 as a predicting system for HCC recurrence was introduced which hope to produce more efficient model of recurrence risk stratification of HCC patients.

## RESULTS

### Baseline characteristics of patients in the training cohort

According to the selection criteria, 448 HCC patients were excluded, and the remaining 198 HCC patients formed the population for the present study. The clinical and tumor characteristics of patients in training cohort are listed in Supplementary Table 1. The total recurrence rate of patients after RR within 24 months was 53.03%. Median time to recurrence was 8 months (range, 1–24 months). The median follow-up period was 16 months (range, 1–89 months).

### Univariate analyses of RFS in the training cohort

RFS was compared for 8 possible prognostic factors, including gender, age, presence of cirrhosis, macroscopic tumor thrombi, microvascular invasion, histological grading, CK19/GPC3 expression pattern and tumor number. Using the Kaplan–Meier method, univariate analysis showed that microvascular invasion (*P*<0.01), macroscopic tumor thrombi (*P*<0.05), moderate vs poor histological grading (*P*<0.01), CK19/GPC3 expression pattern (overall: *P*<0.01; within groups: CK19−/GPC3− vs CK19+/GPC3+: *P*<0.01, CK19−/GPC3+vsCK19+/GPC3+:*P*<0.01) and tumor number (overall: *P*<0.01; within groups both single *vs* two and single *vs* three showed *P*<0.01) were significantly associated with the RFS of patients (Table 1).

**Table 1:** Univariate analysis with respect to tumor recurrence in patients of the training cohort

Characteristic	n	Recurrence-free survival rate (%)			*P* value
		6 months	12 months	24 months	
Gender					P=0.121
Male	158	76.5	61.7	37.4	
Female	40	82.5	64.8	58.8	
Age					P=0.619
≤50	69	78.2	61.8	42.7	
>50	129	77.5	62.6	40.9	
Cirrhosis					P=0.907
Yes	164	78.6	62.4	41.0	
No	34	73.5	61.8	44.3	
Microvascular invasion					P<0.001
Yes	84	65.4	45.8	27.2	
No	114	86.8	74.4	51.8	
Macroscopic tumor thrombi					P=0.024
Yes	6	100	0.0	0.0	
No	192	77.0	63.9	42.4	
Histological grading (tri-classification)					P<0.001
Poorly	88	65.9	49.9	26.6	
Moderately	102	86.2	71.3	50.5	
Well	8	100	85.7	85.7	
Histological grading (bi-classification)					P<0.001
Poorly	88	65.9	49.9	26.6	
Well and Moderately	110	87.2	72.3	52.8	
Immuno-phenotype (tri-classification)					P<0.001
CK19+/GPC3+	38	55.3	41.9	15.4	
CK19-/GPC3+	130	81.5	66.1	46.1	
CK19-/GPC3-	30	90.0	72.0	55.6	
Immuno-phenotype (bi-classification)					P<0.001
CK19+/GPC3+	38	55.3	41.9	15.4	
CK19-/GPC3-and CK19-/GPC3+	160	83.1	67.2	48.1	
Nodule numbers (tri-classification)					P<0.001
1	161	81.3	69.3	48.7	
2	23	65.2	34.8	7.2	
3	14	57.1	28.6	14.3	
Nodule numbers (bi-classification)					P<0.001
1	161	81.3	69.3	48.7	
2 and 3	37	62.2	32.4	10.9	

CK: cytokeratin; GPC3; glypican 3.

In view of the similar RFS of patients between well and moderately differentiated HCC groups, and CK19−/GPC3− andCK19−/GPC3+ groups, and two and three tumor nodules groups, similar parameters within the prognostic factors were combined into the same group. There were high statistical significance in the RFS rates between the groups of the modified bi-classifications of the histological grading, CK19/GPC3 expression pattern and tumor number (well+moderately *vs* poorly: *P*<0.01; CK19−/GPC3+ and CK19−/GPC3−*vs* CK19+/GPC3+: *P*<0.01; single tumor *vs* 2 and 3 tumors: *P*<0.01).

Before performing multivariate analyses, the significant factors in the univariate analysis were assessed for multicollinearity. As showed in the Supplementary Table 2, the VIF values were below the limit of 10 and the tolerance was above the crucial threshold of 0.2 for all the groups which indicated that there was no problematic multicollinearity among the risk factors. Therefore, these factors can be regarded as the major factors and was acceptable for the further analysis.

### Multivariate cox regression analyses and development of recurrence prediction model

**Figure 1:**
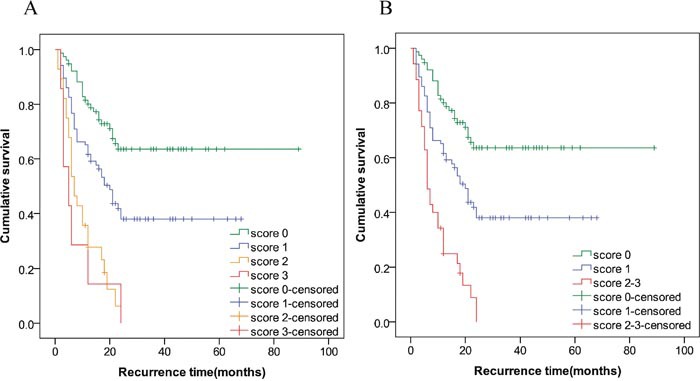
Survival analysis according to the risk factor index The log-rank test showed a significant difference among the survival curves of the four groups (overall: *P*<0.01). However, the survival curve of patients of the score 2 group crossed with that of patients of the score 3 group (*P*>0.05) **(A)**. Then, the patients of the score 2 and 3 were combined and all patients were further subdivided into 3 groups: low risk (score 0); intermediate risk (score 1) and high risk (score 2-3). The log-rank test showed a significant difference among the survival curves of the three groups (Overall: P<0.01; within groups: P<0.01) **(B)**.

The five factors that were significant in the univariate analysis entered in the multivariate analysis. The results showed that CK19/GPC3 expression pattern [hazard ratio (HR)=1.782, 95% confidence interval (CI)=1.137–2.793, *P*=0.012], histological grading (HR=1.969, 95%CI=1.292–3.001, *P*=0.002) and tumor number (HR=2.173, 95%CI=1.328–3.557, *P*=0.002) were the independent predictors of RFS. According to the multivariate Cox regression result, three independent risk factors entered into the multi-factor risk score model. Reference groups were assigned a score of 0. Beta coefficient of poor differentiation was assigned a score of 1 and then Beta coefficient was multiplied by 1.7 (1/0.578) and rounded off to the nearest integer. Then the multi-factor risk index was calculated and all cases were divided into four groups (score: 0, 1, 2, and 3) (Table 2). As shown in Figure 1A, the survival curve of patients of the score 2 group obviously crossed when compared with that of patients of the score 3 group (*P*>0.05). Therefore, the patients were further subdivided into 3 groups: low risk (score 0); intermediate risk (score 1) and high risk (score 2-3). As shown in Table 3, the mean RFS in patients of the low, intermediate and high risk groups were 61.2, 32.6 and 9.6 months, respectively. The log-rank test showed a significant difference among the survival curves of the three groups (*P*<0.01) (Figure 1B). The estimated survival rate of patients in the high, intermediate and low risk groups at 24 months were 0, 38.1%, and 63.6%, respectively.

**Table 2:** Multivariate Cox regression analysis and integer score assignment algorithm based on the β-coefficients

Variable	B	HR	95.0% CI (lower-upper)	P value	Score
**CK19/GPC3 expression**					
CK19-/GPC3-and CK19-/GPC3+	0	reference		0.012	0
CK19+/GPC3+	0.578	1.782	1.137-2.793		1
**Histological grading**					
Well and moderately	0	reference		0.002	0
Poorly	0.678	1.969	1.292-3.001		1
**Nodule number**					
Single	0	reference		0.002	0
Two or three	0.776	2.173	1.328-3.557		1
**Microvascular invasion**	0.303	1.353	0.874-2.096	0.175	
**Macroscopic tumor thrombi**	0.402	1.495	0.579-3.857	0.406	

HCC: hepatocellular carcinoma; HR: Hazard ratio; CI: confidence intervals; CK: cytokeratin; GPC3: glypican 3.

**Table 3:** Recurrence-free survival by the risk score in patients of the training cohort

Group	n	Estimated rates of RFS (%)			Mean of RFS(months)			*P*
		6 month	12 month	24 month	95.0% CI (lower-upper)	Estimate	*Std*. error	
**Score 0**	77	92.1	80.1	63.6	52.336-70.054	61.2	4.520	
**Score 1**	86	76.7	61.6	38.1	26.175-39.112	32.6	3.300	<0.001
**Score 2-3**	35	48.6	24.9	0.00	7.090-12.031	9.6	1.260	

RFS: recurrence-free survival ; CI: confidence Intervals; *Std*. Error: standard error.

### Comparing the performance of the risk score model with the other factors in the training cohort

**Figure 2:**
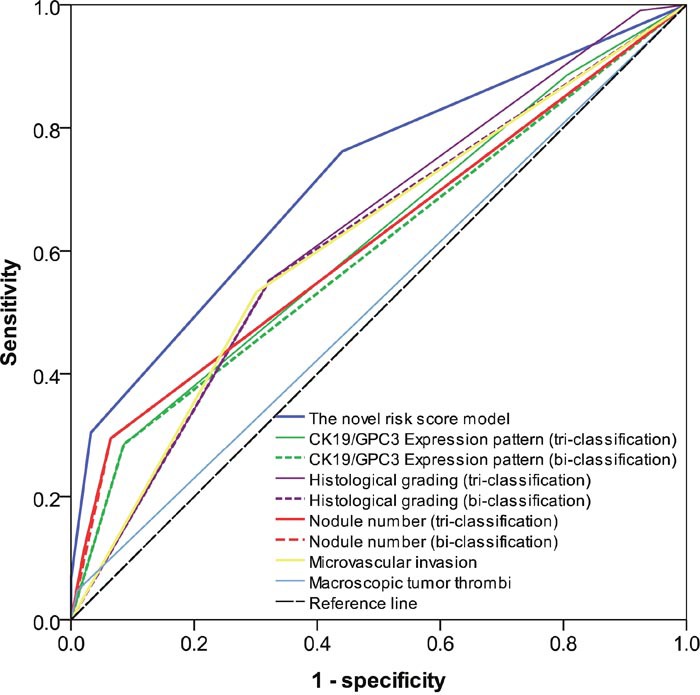
Overall predictive performance was measured by AUC of the receiver-operating characteristic curve The AUC value of the novel risk score model was the highest comparing to the other factors.

The ability of the novel risk score model and other factors to predict tumor recurrence was shown in Figure 2. AIC identified the novel risk score model as the optimal one (it displayed the lowest AIC value, 1067.707), in agreement with the highest AUC (0.715, 95%CI= 0.645 to 0.786, P<0.001) (Table 4).

**Table 4:** AIC and AUC of the risk score model and other factors in patients of the training cohort

Score model and other elements	AIC value	AUC value(95% CI)	*P*
**Multi-factor risk score model**	1067.707	0.715(0.645-0.786)	<0.001
**CK19/GPC3 expression pattern** (tri-classification)	1095.187	0.617(0.539-0.694)	=0.005
**CK19/GPC3 expression pattern** (bi-classification)	1094.638	0.600 (0.521-0.678)	=0.015
**Histological grading** (tri-classification)	1086.532	0.629 (0.551-0.706)	=0.002
**Histological grading** (bi-classification)	1090.403	0.615(0.536-0.693)	=0.005
**Nodule number** (tri-classification)	1097.820	0.616 (0.538-0.694)	=0.005
**Nodule number** (bi-classification)	1095.108	0.615 (0.538-0.693)	=0.005
**Microvascular invasion**	1093.547	0.616(0.538-0.694)	=0.005
**Macroscopic tumor thrombi**	1100.316	0.518(0.438-0.599)	=0.655

AIC: Akaike information criterion; AUC: area under roc curve; CI: confidence intervals; CK: cytokeratin; GPC3: glypican 3.

### Confirmation of prognostic ability of the model in the validation cohort

**Figure 3:**
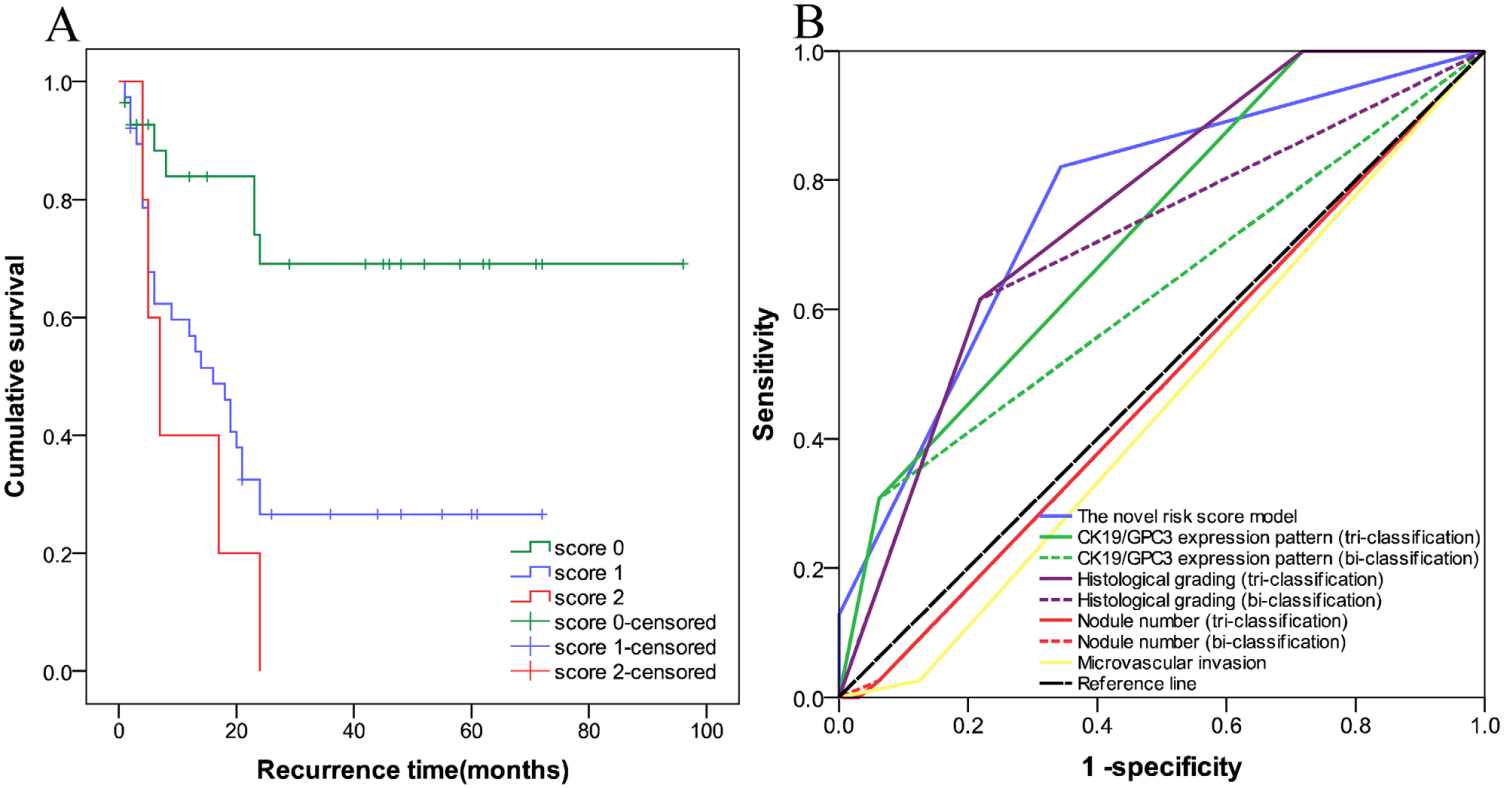
The prognostic ability of the recurrence prediction model was validated in an independent validation cohort In **(A)**, the log-rank test demonstrated a significant difference between the survival curves of the three groups (P<0.001). **(B)** Showed that AUC value of the risk score model was the highest comparing to that of the other factors in the validation cohort.

The characteristics of the validation cohort were summarized at Supplementary Table 3. The median RFS time was 18 months (range, 1–96 months). With the median recurrence time of 7 months (range, 1–24 months), the recurrence rate in the validation cohort was 54.9%. One (1.4%) patients from this cohort died during the follow-up without metastasis or recurrence. As shown in Figure 3A, the log-rank test demonstrated a significant difference between the survival curves of the three groups (P<0.001). Within the 24 months after RR, the estimated rate of RFS of patients was 69.1% in score 0 group and 26.6% in score 1 group. And all patients in score 2 group underwent recurrence within the 2 years after RR. The mean RFS time in score 0, 1 and 2 groups were 70.5, 27.0 and 11.4 months, respectively. A significent difference in mean RFS time between the three groups were found (Table 5). The novel model showed high performance with a good discrimination ability in the validation cohort. In the validation cohort, the AUC value of the novel risk score model was 0.760 (95%CI=0.647−0.874), which was the highest among all the factors and surpassed that of the training cohort (Figure 3B).

**Table 5:** Recurrence-free survival by the risk score in patients of the validation cohort

Group	n	Estimated rates of RFS (%)			Mean of RFS(months)			*P*
		6 month	12 month	24 month	95.0% CI (lower-upper)	Estimate	*Std*. error	
**Score 0**	28	88.3	83.9	69.1	54.598-86.410	70.5	8.115	
**Score 1**	38	62.3	56.9	26.6	17.976-36.048	27.0	4.610	<0.001
**Score 2-3**	5	60.0	40.0	0.00	3.743-19.057	11.4	3.906	

RFS: recurrence-free survival ; CI: confidence Intervals; *Std*. Error: standard error.

## DISCUSSION

Most HCC patients could be detected at early stages using imaging technology. With the application of MC or Barcelona Clinic Liver Cancer (BCLC) staging system [17], the tumor recurrence rates after liver transplantation (LT) or RR was significantly reduced. LT has been proved to be the optimal therapy for HCC patients at early stage, but due to the shortage of liver donors, patients choose RR. Therefore, discriminating the patients with high risk of recurrence and establishing a set of optimal criteria for early stage HCC patients are crucial. In the present study, 198 HCC patients who received RR and conformed to MC were investigated. Regression analysis showed that three risk factors including CK19/GPC3 expression pattern, histological grading and multiple tumors were regarded as the independent predictors for HCC recurrence. Results of the Cox regression devised a risk predicting model which sub-classified HCC patients into high, intermediate and low risk groups. Overall results indicated that the model can effectively stratify the risk of recurrence of HCC patients fulfilling MC after RR.

During the past decade, studies aiming at identifying the risk factors for predicting the recurrence in HCC patients who underwent RR were numerous. Many studies have similarly emphasized the significance of tumor diameter and number, poor differentiation, micro-vascular invasion and the presence of macroscopic tumor thrombi in risk of recurrence in HCC according to the MC after liver resection. However, as independent predictors, the importance of these factors varied depending on their probability weight in different recurrence predicting models [18, 19, 20]. In the present study, although micro-vascular invasion and the presence of macroscopic tumor thrombi were significantly associated with RFS of patients but results from Cox regression implied that other factors remained in the model have more and strong predicting capacity for recurrence.

Many other models were designed by introducing appropriate biomarkers such as serum AFP, HBsAg, cytoskeleton-associated protein 2, plasma fibrinogen which showed improved prognostic power for tumor recurrence and provided a wider perspective to consider for RR indication [21, 22, 23, 24, 25]. However, limited models studied HCC in the early stages and hence more evidences were necessary to highlight the importance of these promising biomarkers and models in the risk stratification for recurrence in future.

In our previous study, which included extra 120 HCC patients beyond MC who underwent RR were included, significant differences in the RFS rates were observed between the patients of the CK19+/GPC3+ and CK19−/GPC3+ or CK19−/GPC3− groups [12]. However, in another cohort of HCC patients fitting MC who underwent LT in our center showed significant differences in RFS rates and were placed between the three CK19/GPC3 groups [26]. In the current cohort of HCC patients within MC who underwent RR, only CK19+/GPC3+ phenotype HCC was significantly associated with the recurrence and was consistent with that of our previous study [12]. These results implied that CK19+/GPC3+ expression can be a useful indicator for recurrence prediction in patients within or beyond MC when RR was selected. In addition, the risk of recurrence should be evaluated separately during RR or LT in HCC patients with the same CK19/GPC3 expression pattern.

Majority early recurrence of HCC after treatment was due to the dissemination of the primary tumor. Furthermore, dissemination ability of tumor was determined by the aggressive biological properties of tumor cells. The prognostic indication of CK19 or GPC3 expression in HCC had been largely investigated previously, whereas in molecular level, there is little evidence to clarify the relationship between CK19 or GPC3 expression and aggressive biology of this neoplasm. Somatic mutations are the most common phenomenon in tumorigenesis, however, in HCC, the somatic mutations of *ck19 or gpc3* genes or their modulators were rarely reported.

During the differentiation of hepatoblast/hepatic progenitor cells towards the mature hepatocyte, the expression profiling of CK19/GPC3 converts unidirectionally. Therefore, it is easy to speculate that different immune-subtypes of HCC could originate from the hepatic parenchyma cells of different differentiation stages and have been kept the labels of specific CK19/GPC3 expression profiling as their normal counterparts. The homing potential is one of the most important features of stem cell. Previous literatures revealed that regulated migration of stem cells is critical for organogenesis during development and tissue repair in adulthood. In addition, the migration ability between stem cells and their differentiated progenies are of great difference [27]. In terms of this analogy, more frequent intrahepatic metastasis, microvascular invasion, regional lymph node involvement, and distant metastasis of CK19+/GPC3+ HCC seem to be a reflex of a stronger migration ability of the transformed hepatoblast/hepatic progenitor cells. If the hypothesis were true, it could be concluded that a more aggressive biological behavior of tumor cells with the stamp of CK19 or GPC3 positive expression can be inherited from their tumor initiating cells, which could have been determined by the epigenetic modulations during the differentiation process before malignant transformation. More evidence is needed to clearly support the hypothesis in future.

Combing CK19/GPC3 sub-typing, histological grading and tumor number into MC, the novel model significantly improved the stratification for early recurrence in patients fulfilling the MC after RR. At 12 and 24 months after RR, estimated rates of RFS in the patients with score 0 were 80.1% and 63.6%, respectively, which were higher than that of the average [1, 2]. Thus, these patients can be eligible for RR. The estimated RFS rates in patients with score 1 were 61.6% and 38.1% at 12 and 24 months after RR, respectively, which was close to that of the average level. Therefore, when RR was performed, patients with score 1 should be followed up closely with continued surveillance for recurrence. The risk of recurrence in patients with scores 2 or 3 was very high when RR was performed. Once RR was selected, patients in this group should be strictly monitored so as to find the recurrence or metastasis timely.

Needle biopsy is a useful and common method in the diagnosis of the lesions of the liver [28]. Combining the results of IHC and imaging technologies, it could be found that the CK19/GPC3 expression pattern, tumor number and size, as well as histological grading degrees could be retrieved, which indicated that the novel risk score model could be potentially used in the therapeutic decision making. Since subsets of patients with scores 1 or 0 showed equivalent or fewer recurrence rates compared to the patients fulfilling MC, RR can be an acceptable method for these patients through close surveillance. For patients with scores 2 or 3, therapeutic effect would be challenged by high recurrence when RR was performed and LT might be an alternative choice. However, tumor recurrences after LT of these patients need to be further investigated.

This study has some limitations. The first limitation included its retrospective nature and associated issues including patient selection bias, which may challenge the practical utility of our model because interventional therapy before RR was usually conducted for patients with HCC even fulfilling MC in some transplantation center. Secondly, this study was conducted at only two centers and a large sample size of this tumor is needed in future to establish a comprehensive model. Thirdly, the consistency in the CK19/GPC3 IHC staining between core needle biopsy and surgical specimens need further investigations to generate high-level medical evidence.

## MATERIALS AND METHODS

The nature of this study was fully explained to these patients, and informed consent was obtained from them before enrollment. The study protocol was accorded ethically with the principles of Declaration of Helsinki (1983) and was approved by the Ethical Committee of Beijing You-An Hospital, Capital Medical University.

### Study design

**Figure 4:**
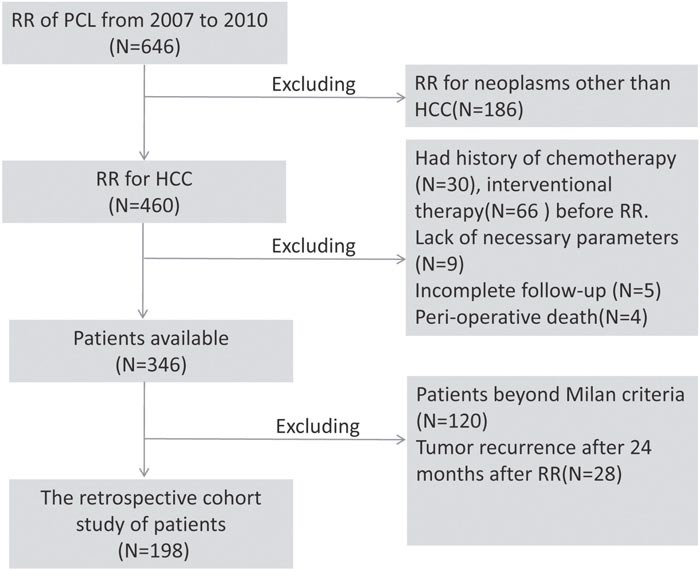
Flow chart of patient selection procedures RR: radical resection; PCL: the primary carcinoma of the liver; HCC: hepatocellular carcinoma.

Between January 2007 and October 2010, a total of 646 patients with HCC underwent RR at You'an Hospital, Capital Medical University, and 304 Hospital, PLA, Beijing, P.R. China, and their records were reviewed. According to the selection criteria (Figure 4), 448 patients with HCC were excluded, and the remaining 198 patients with HCC formed a training dataset to develop a prognostic model for predicting recurrence. Based on the same selection criteria, another independent validation cohort of 71 patients from January 2004 to December 2006 at You'an Hospital, Capital Medical University was used to assess the performance of the model.

All surgeries were performed by the same group of doctors, with resection margin >2 cm. Milan criteria were defined as solitary tumor ≤5 cm; or ≤3 tumor nodules with the largest diameter ≤3 cm, and absence of vascular invasion or metastatic invasion using computed tomography. Other entry criteria for RR included: (1) the entire tumor (including the main tumor, satellites and multicenter tumors) was resectable; (2) liver function was classified as grade A or B of the Child–Pugh classification; (3) the remnant volume of liver was considered adequate and no contraindication to operation was found. In our center, if patients conformed to MC, no other anticancer treatment was given to the patients after RR until recurrence.

### Follow-up

All patients were followed up at the outpatient clinic every month from the date of initial treatment till January 2015, or till death. During follow-up visits, patients were subjected to routine examinations such as abdominal and pelvic ultrasonography and chest X-rays. Recurrence was defined as the appearance of new lesions with radiological features typical of HCC, seen by at least two imaging methods by contrast-enhanced computed tomography or magnetic resonance imaging scan and, if necessary, by ultrasound-guided biopsy to confirm the diagnosis.

### Immunohistochemical stains

The immunohistochemistry (IHC) was performed as described previously [29]. Mouse anti-human GPC3 monoclonal antibody (Clone 1G12; Dilution, 1:200) and mouse anti-human CK19 monoclonal antibody (Clone BA17; Dilution, 1:100) were purchased from Zeta company (Sierra Madre, CA, USA). Steamer for 20 minutes in citrate target retrieval buffer (pH 6.0). The results of immunohistochemical staining were considered positive if greater than 5% of the tumor cells showed cytoplasmic staining for GPC3 or CK19. The cases were divided into three groups: CK19+/GPC3+ group, included cases where tumor cells co-express GPC3 and CK19; CK19−/GPC3+ group, included cases where tumor cells express GPC3 singly; and CK19−/GPC3− group, included cases with negative expression of both GPC3 and CK19. Yolk sac tumor tissue was used as a positive control sample for GPC3. The evidence of cytoplasmic staining of adjacent interlobular duct epithelia served as internal positive control for CK19. Negative controls were carried out by substitution of the primary antibodies with non-immunized serum, which resulted in no signal detection. The expression of these markers was assessed independently and blindly by two investigators. All slides were reviewed to confirm the diagnosis according to the guidelines of World Health Organization (WHO) criteria, 2010 [30]. The result of pathology in each specimen all report complete resection and resection margin>2cm according to the definition of radical resection in this study.

### Statistical analysis

All analyses were performed using the SPSS (version 21.0, SPSS, Chicago, IL). Categorical data were described by frequency and percentage, whereas continuous data by mean±SD or median (range). χ2 test and Student *t* test were applied to compare the distribution of categorical and continuous variables, respectively. The recurrence free survival (RFS) time was calculated from the date of operation till the date of first distant or local disease recurrence or the last follow-up. Patients who died before experiencing disease recurrence were considered censored. Survival curves for the patients were plotted using the Kaplan-Meier method and differences between the curves were assessed using the log-rank test. Univariable and multivariable Cox proportional hazard regression analysis was used to assess factors associated with recurrence of HCC. Before performing multivariate analyses, the significant factors in the univariate analysis were assessed for multicollinearity. A tolerance of less than 0.2 and/or a variance inflation factor (VIF) of 10 and above indicated a multicollinearity problem [31, 32]. A summary risk score for tumor recurrence was derived from the β coefficients associated with the significant predictor variables in the final selection model for tumor recurrence. A risk score for each variable based on its coefficient value, standardized with the lowest value, which was assigned a value of 1, and rounded to the nearest integer. The summary risk score for an individual was obtained by summing up the weighted scores of each of the risk factors. The overall predictive performance was measured by AUC of the ROC curve in both training and validation cohorts, with 0.5 and 1.0 indicating no and perfect predictive ability, respectively. The Akaike information criterion (AIC) was used to compare the performance of the factor risk score model [33]. *P*<0.05 was considered statistically significant.

## CONCLUSION

Combing CK19/GPC3 sub-typing, histological grading and tumor number into MC, a model to stratify the risk of recurrence of HCC patients after RR was devised. The model could be used to predict the recurrence when RR was performed. In addition, by combining the core needle biopsy, IHC and imaging technologies, the model could be potentially used to guide therapeutic decisions before RR.

## SUPPLEMENTARY MATERIALS TABLES



## Abbreviations

HCC: hepatocellular carcinoma
RR: radical resection
MC: the Milan criteria
HPC: hepatic progenitor cell
CK: cytokeratin
GPC3: glypican 3
IHC: immunohistochemistry
RFS: recurrence-free survival
AIC: Akaike information criterion
VIF: variance inflation factor
CI: confidence intervals
HR: hazard ratio
AUC: area under the curve
