# Supplementary material for: Prediction of early recurrence of hepatocellular carcinoma within the Milan criteria after radical resection
Source: Oncotarget. 2017 Jun 28;8(38):63299–310. doi: 10.18632/oncotarget.18799 (PMC5609922; doi:10.18632/oncotarget.18799)
Supplement: Supplementary file 1 [file oncotarget-08-63299-s001.pdf]

Prediction of early recurrence of hepatocellular carcinoma within the Milan criteria after radical resection

SUPPLEMENTARY MATERIALS

Supplementary Table 1: Baseline clinical characteristics of patients in the training cohort

Variables		Value
**Age, years**	Median	55
	Range	17-80
	Mean±SD	53.9±10.7
**Male, n(%)**		158(79.8)
**Cirrhosis, n(%)**	Yes	164(82.8)
**Etiology, n(%)**		
	HBV infection	183(92.4)
	HCV infection	12(6.1)
	Alcohol abuse	1(0.5)
	Primary biliary cirrhosis	1(0.5)
	HCV infection + Alcohol abuse	1(0.5)
**Immuno-phenotype, n(%)**		
	CK19+/GPC3+	38(19.2)
	CK19-/GPC3+	130(65.7)
	CK19-/GPC3-	30(15.2)
**Number of tumor nodule, n(%)**		
	single	161(83.3)
	two	23(11.6)
	three	14(7.1)
**Tumor burden**		
**Diameters of a single tumor(cm)**	Median	2.7
	Range	0.5-5.0
	Mean±SD	2.8±1.1
**Cumulative diameters of 2 or 3 tumors(cm)**	Median	3.6
	Range	0.9-7.0
	Mean±SD	3.7±1.3
**Histological grading, n(%)**		
	Poorly	88(44.4)
	Moderately	102(51.5)
	Well	8(4.0)
**Microvascular invasion, n(%)**	Yes	84(42.4)
**Macroscopic tumor thrombi, n(%)**	Yes	6(3.0)

SD: standard diviation; HBV: hepatitis B virus; HCV: hepatitis C virus; CK19: cytokeratin 19; GPC3: glypican 3.

Supplementary Table 2: Multicollinearity analysis

Variable	Adjusted R^2	Unstandardized coefficients		Standardized coefficients	t	*P*	Tolerance	VIF
		B	*Std.* Error	Beta				
**CK19/GPC3 expression pattern** (bi-classification)	0.118	-5.978	2.833	-0.147	-2.110	0.036	0.927	1.079
**Histological grading** (bi-classification)		-7.363	2.381	-0.228	-3.092	0.002	0.824	1.213
**Nodule number** (bi-classification)		-5.651	3.030	-0.137	-1.865	0.064	0.828	1.208
**Microvascular invasion**		-2.113	2.493	-0.065	-0.847	0.398	0.760	1.316
**Macroscopic tumor thrombi**		-7.967	6.469	-0.085	-1.232	0.220	0.939	1.065

Dependent Variable: recurrence; VIF: variance inflation factor; *Std.* Error: Standard error; CK: cytokeratin; GPC3: glypican 3
A tolerance of less than 0.20 and/or a VIF of 10 and above indicates a multicollinearity problem.

Supplementary Table 3: Baseline clinical characteristics of patients in the validation cohort

Variable		Value
**Age, years**	Median	62.0
	Range	43-87
	Mean±SD	61.8±1.01
**Male, n(%)**		61(85.9)
**Cirrhosis, n(%)**	Yes	66(93.0)
**Etiology, n(%)**		
	HBV infection	65(91.5)
	HCV infection	4(5.6)
	Alcohol abuse	1(1.4)
	Primary biliary cirrhosis	1(1.4)
**Immuno-phenotype, n(%)**		
	CK19+/GPC3+	9(12.7)
	CK19-/GPC3+	48(67.6)
	CK19-/GPC3-	14(19.7)
**Number of tumor nodule, n(%)**		
	1	68(95.8)
	2 or 3	3(4.2)
**Tumor burden**		
**Diameters of a single tumor(cm)**	Median	2.5
	Range	1.0-5.0
	Mean±SD	2.7±0.1
**Cumulative diameters of 2 or 3 tumors(cm)**	Median	3.5
	Range	3.5-3.9
	Mean±SD	3.6±0.1
**Histological grading, n(%)**		
	Poorly	31(43.7)
	Moderately	31(43.7)
	Well	9(12.7)
**Microvascular invasion, n(%)**	Yes	5(7.0)
**Macroscopic tumor thrombi, n(%)**	Yes	0(0.0)

SD: standard diviation; HBV: hepatitis B virus; HCV: hepatitis C virus; CK19: cytokeratin 19; GPC3: glypican 3.
